# Scene Acquisition with Multiple 2D and 3D Optical Sensors: A PSO-Based Visibility Optimization

**DOI:** 10.3390/s20061726

**Published:** 2020-03-19

**Authors:** Francesco Buonamici, Rocco Furferi, Lapo Governi, Antonio Marzola, Yary Volpe

**Affiliations:** Department of Industrial Engineering of Florence, University of Florence, Via di S. Marta 3, 50139 Firenze, Italy; rocco.furferi@unifi.it (R.F.); lapo.governi@unifi.it (L.G.); antonio.marzola@unifi.it (A.M.); yary.volpe@unifi.it (Y.V.)

**Keywords:** visibility analysis, optical sensors, 3D scanning, computer graphics, PSO, body scanner, sensor placement

## Abstract

Designing an acquisition system for 2D or 3D information, based on the integration of data provided by different sensors is a task that requires a labor-intensive initial design phase. Indeed, the definition of the architecture of such acquisition systems needs to start from the identification of the position and orientation of the sensors observing the scene. Their placement is carefully studied to enhance the efficacy of the system. This often coincides with the need to maximize the surfaces observed by the sensors or some other metric. An automatic optimization procedure based on the Particle Swarm Optimization (PSO) algorithm, to seek the most convenient setting of multiple optical sensors observing a 3D scene, is proposed. The procedure has been developed to provide a fast and efficient tool for 2D and 3D data acquisition. Three different objective functions of general validity, to be used in future applications, are proposed and described in the text. Various filters are introduced to reduce computational times of the whole procedure. The method is capable of handling occlusions from undesired obstacle in the scene. Finally, the entire method is discussed with reference to 1) the development of a body scanner for the arm-wrist-hand district and 2) the acquisition of an internal environment as case studies.

## 1. Introduction

Visibility analysis is a crucial task that can be used to solve a wide range of problems in different fields [1,2]. To cite a few, visibility analysis is essential to decide the position of watchtowers to monitor fires in forest areas [3], to identify the best position of optical sensors in product inspection [4], to carry out environment impact assessment (e.g., planning of the position of structures, power lines, etc.) [5], to plan operations in both military applications [6] and history studies [7].

While its usefulness in different areas is undisputed, visibility analysis can still be improved and tuned to confront with artificial vision applications. In fact, visibility analysis usually does not consider any limitation in the Field of View (FOV), hypothesizing an observer free to rotate around the observation point. Dealing with fixed sensors, on the other hand, is important to accurately model the specific features and performances of the available hardware in order to provide a reliable result.

In other words, considering the acquisition of 2D and 3D information on a scene through optical sensors, it is essential to carefully study their optimal position and orientation (i.e., extrinsic camera parameters) taking into account the specific features and performances of the acquisition device to assure reliable and accurate results.

When the Machine Vision (MV) system is designed to perform a one-time acquisition of an object, visibility analysis is usually not a challenge since it is possible to acquire data from different points until the entire subject is covered and redundant data may be collected. This allows to: i) increase the statistical validity of data, ii) tolerate possible mistakes and errors in the acquisition, and iii) obtain data that can be easily registered. In this scenario, the experience and skills of the user help identifying the positions that minimize the number of required acquisitions and increase the quality of the obtained data, hence speeding up the process and increasing its efficacy. 

Often, however, it is necessary to design a system that needs to continuously acquire a scene and/or to perform multiple acquisition of similar objects. In this case, the definition of the position of the sensor(s) is essential to allow a correct acquisition of the scene and to minimize possible occlusions. Since the extrinsic parameters of the sensor(s) will remain fixed, a wrong choice of the position(s) will heavily affect the effectiveness of the system during its service life. The design of optical 3D scanner based on stereo vision or triangulation dedicated to the acquisition of a specific class of objects or geometries is a valid example. In this case, the number, and intrinsic/extrinsic parameters of sensors need to be carefully studied to produce an effective system. Similarly, in case of fixed 2D cameras observing a scene (as in surveillance systems) it is important to choose the perfect location to frame the area of interest while reducing optical occlusions. 

In all these cases the execution of physical tests, dedicated to the identification of the advantageous type of sensor and their position and orientation, although useful, imposes significant times and costs. An alternative strategy relies on virtual simulations and analyses to identify if and how a part of a given scene is visible for a sensor. This strategy requires a digital 3D model of the scene to be used as reference during the process; this can be only an interpretation of the object to acquire (e.g., a coarse model) and not a detailed 3D model of the scene. Such a model can be acquired by means of any 3D acquisition technology or even modeled within a CAD software package to build a schematic and faithful representation of the physical geometry. Different methods and algorithms can be used to compute if a part of a surface or a single point belonging to the scene is visible for an optical sensor. Most methods rely on a pinhole description of the optical sensor and perform some kind of line-of-sight analysis to assess points visibility, e.g., Ray Tracing analysis (RT). 

Another widely known method is the so-called “viewshed” analysis used in geographic information systems. In this context, it is fundamental to evaluate the geographical area that is visible from a location. The above-mentioned technique uses the elevation value of each cell of a discrete global grid with an associated height map to determine visibility to or from a query point. This allows for efficient computations but poses some limitations on the possible applications of this class of algorithms.

Unfortunately, the variables of the virtual visibility analysis problems are typically user-guided. In fact, the overall architecture of the MV system variables is usually defined by a trial-and-error procedure starting from a first hypothesis produced by the user. Through continuous slight changes of the variables, such an initial hypothesis is edited until the best possible configuration is reached. Occasionally, configurations that differ significantly are tested but the main contribution always comes from the user. This approach poses some limits on the number of configurations that can be tested and, more importantly, the global search for the best configuration is entirely determined by the user’s experience. 

An automatic optimization procedure could bring a good contribution to the design phase, allowing the execution of multiple analyses in a small-time window and, most importantly, the seeking of the best possible configuration, not rigidly bounded to any pre-assumption. This is a situation similar to the most famous “art gallery problem” of the computational geometry field [8]. Some approaches were proposed in the state of the art [1,9] dealing with automatic optimization procedures. However, such methods are mostly addressed to design 2D acquisition systems and, to best of author’s knowledge, no procedure to address the virtual visibility analysis of 3D sensors has been carried out. 

This is a shame since, considering the development of body scanners and similar acquisition devices for anatomical regions, an automatic optimization procedure would significantly improve the design process. Up to this moment, cameras are manually placed by designers, who usually prefer simple solutions (i.e., regular distribution patterns as in [10,11]) rather than experimenting with innovative solutions. In rare cases, an experiment is carried out to identify the most valid solution among a finite number of alternatives as in [11]; even in this recent work, however, tested solutions are essentially regular patterns with different spacing values. While a regular pattern would be a good choice if the object to be acquired has a regular shape, it would be better to seek a sensor distribution pattern that keeps into consideration the geometry of shapes that need to be acquired.

Accordingly, the present paper proposes an automatic optimization method for the identification of the best configuration of optical sensors acquiring a scene. The method relies on data obtained on a series of visibility analyses performed on a 3D model of the scene to identify the most convenient setting (extrinsic parameters) according to a custom-built metric. Possible application of the procedure might be: i) the design of 2D surveillance camera systems based on an arrays of cameras, ii) the development of 3D scanners based on multiple acquisition points specifically tuned to acquire a class of known objects, iii) geographical systems, where the identification of the best positions to place optical sensors of any kind is fundamental; with small modification the method could be extended to antennas and other kind of emitters whose efficiency is influenced by line-of-sight. 

Due to the strong interest in the setup of 3D acquisition devices, in this work the devised method was tested on the design of a multi-camera 3D scanner for the acquisition of the anatomical arm-wrist-hand district of pediatric patients. The specific application required the maximization of the area visible by a set of RGB-D cameras used while imposing a certain amount of overlapping region useful for subsequent post-processing data operations. Moreover, the procedure was tested on a coarse model obtained from a 3D scanning of an indoor environment to assess the generality of the procedure. 

## 2. Materials and Methods

With the aim of devising a method for optimizing the visibility of optical devices, the following requirements were defined:

- Use of a highly compatible 3D format as reference for the scene (e.g., Standard Tessellation Language STL format)
- Accurate modelling of optical sensors (both 2D and 3D sensors i.e., RGB and RGB-D cameras)
- Optimization parameters considered by the procedure are extrinsic parameters
- GUI allowing a direct visualization and navigation of the scene and the results, highlighting which surfaces are visible to every single sensor
- Good speed performances, allowing to run the optimization on models characterized by a high level of detail and corresponding high number of triangles.
- A range of valid objective functions guiding the optimization towards different results; these should cover typical situations that could be of interest for the user in specific applications (e.g., maximize surface area, number of triangles, overlapping regions, consider occlusions from obstacles that are not of interest).

**Figure 1:**
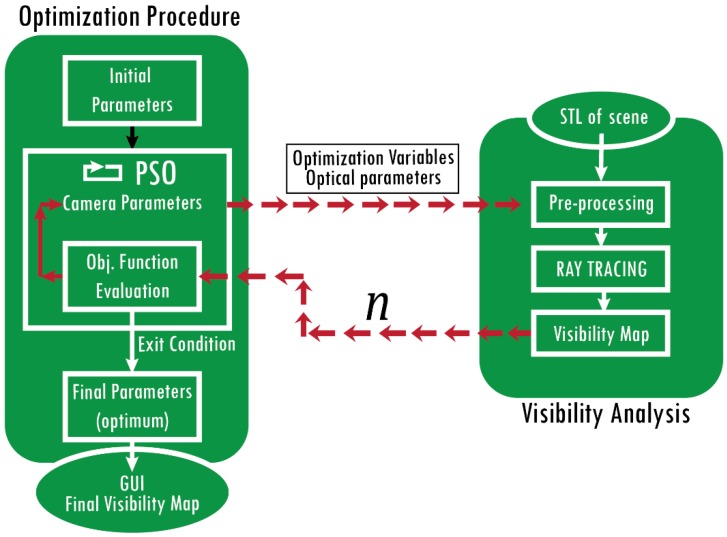
Framework of the method: optimization procedure and visibility analysis.

Starting from these premises, it is possible to outline a basic functional structure for the method. As depicted in Figure 1, the whole method consists of two steps: a *visibility analysis* and an *optimization procedure*. The core element of the method consists of an algorithm able to evaluate if a single triangle of the STL model, used as a reference of the scene, is visible by a sensor (visibility analysis). In doing so, the algorithm considers extrinsic parameters of the sensor and intrinsic parameters (viewing frustum, maximum and minimum distance of acquisition). Each triangle of the model is then classified as visible or not in order to build a Visibility Map (VM) of the entire scene for each sensor considered in the analysis. Subsequently, these “raw” data are used to build an objective function to be minimized to find the optimal solution. The objective function is iteratively evaluated for each set of plausible variables within an optimization procedure in order to find the global optimum. 

In the presented implementation, a RayTracing algorithm (RT) is used as baseline element to assess the visibility of each STL triangle. In its most common implementation, the RT algorithm computes the intersection between rays that pass through the camera optical center and each “pixel” of the image plane to identify the point of the scene that is “hit” by the ray. As a result, by applying a series of rays covering the entire FOV of the sensor (i.e., sweeping the entire image plane), it is possible to identify all the surfaces observed by the sensor. A GPU (Graphics processing unit) implementation of the classic RT algorithm is used in this study to speed up the process. From an optimization perspective, the problem faced is not characterized by an explicit functional that can be minimized; moreover, the shape of the solution space is evidently non-convex and with multiple local minima. Accordingly, a heuristic approach is the most suited to try to reach the global optimum. The Particle Swarm Optimization (PSO) is the elective algorithm [12] to guide such a process. As widely known, the PSO is a population-based algorithm that tests, for each iteration, a set of possible solutions (i.e., population), updated at each iteration according to the best values found by the population.

Considering the input/output flow of Figure 1, the optimization procedure starts with a set of initial extrinsic parameters of the sensors used in the analysis. This set is used to initialize the optimization and should be provided by the user. A plausible starting set is the one where the sensors frame different areas of the subject. Considering n sensors observing the scene, each evaluation of the objective function corresponds to n calls to the visibility analysis. 

The input of this analysis are:

- The optimization variables (i.e., extrinsic parameters) values, which may be different for each call;
- The intrinsic parameters (i.e., FOV angles, maximum and minimum acquisition distances), which are usually fixed during the optimization, as they depend only to the choice of a sensor model used in the application.

Once the camera model is correctly updated and introduced in the scene, the RT analysis allows the computation of the VM. The objective function, whose possible formulation is described in the following, receives the contributes from each camera, compares the results to account for overlapping regions or other global effects (e.g., quality of observation, surfaces that are always hidden), and produces a numerical value describing the quality of the tested solution. Whenever a stopping criterion is met, the final set of optimization variables is used to build the final global VM. This represents the principal result of the entire analysis. An appositely devised GUI is provided to the user in order to navigate the scene and to assess which areas are visible. 

Both the visibility analysis and the optimization procedures are detailed in the following sections.

### 2.1. Visibility Analysis

As mentioned above, the visibility analysis is called every time that the method needs to evaluate which part of the scene is visible for a single camera. It receives the optimization variables and the intrinsic parameters of the sensor as input and returns a map of the STL triangles that are visible (VM). The optimization variables describe the extrinsic parameters of the sensor, while the intrinsic parameters are required to faithfully simulate the performances of the sensor used. Summing up, the entire camera pinhole model used in the analysis is controlled by these parameters:

- Optimization variables—extrinsic parameters (which change at every call of the visibility analysis)
  - Camera position (*X*, *Y*, *Z* coordinates of the camera optical center). XYZ coordinates are defined in the reference frame of the scene model.
  - Camera orientation (*X*, *Y*, *Z* components of a unit vector that defines the optical axis of the camera)
  - Roll angle (defined by the rotation of the image plane around the Camera Orientation vector)
- Intrinsic parameters
  - Viewing frustum—the method uses a rectangular viewing frustum characterized by two angles (vertical and horizontal aperture angles)
  - maxD, minD —maximum and minimum distance for the acquisition—while technically the entire frustum is visible for a camera, each type of sensor can be characterized by a minimum and maximum distance of acquisition. As an example, RGB cameras could be affected by a limited depth of focus; similarly, RGB-D cameras and generally, 3D optical scanners, are able to acquire data only within a certain depth window.

While the situation described above is the one adopted by the present work, depending on the specific application, some changes could be introduced in the optimization variables. Some parameters could be included among the optimization variables in a controlled way; as an example, the optimization could use binary values to decide which of two sensors with different performances (i.e., intrinsic parameters) is preferable for an application. 

The strive for high speed VM computation brought to the development of a series of pre-processing steps (see Figure 1) to “lighten” the reference 3D model for subsequent operations. It is important to note that the RT algorithm cost is directly influenced by the number of polygons that need to be evaluated. Accordingly, a reduction of the number of triangles that need to be processed by the RT algorithm is the most influencing factor for the minimization of the computation time. This can be done by i) decimating the reference model at cost of a quality loss, or by ii) removing the areas that, given a certain configuration, can be excluded a priori from the analysis. 

On a practical level, a series of filters can be applied to pursue the second strategy without recurring to a proper RT analysis. Depending on the specific conditions, this can heavily reduce the number of triangles to be processed by the RT.

#### 2.1.1. Pre-Processing

**Figure 2:**
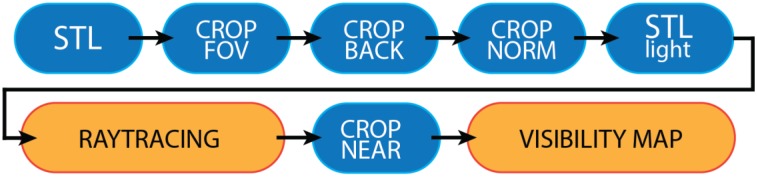
Visibility analysis framework: filters applied to the 3D model in order are depicted in blue, the main elements of the procedure (Ray Tracing analysis and the desired result—the Visibility Map (VM)) are depicted in orange.

Specifically, the scene model is filtered with consecutive filters (See Figure 2 for an expanded diagram of the steps of the visibility analysis phase) that remove unnecessary part of the model that are certainly hidden to the sensor (each filter, implemented within Matlab® environment, is later described in detail):

(1) *CropFOV*—removes the points that do not fall inside the FOV volume of the camera.
(2) *CropBack*—removes all backfaced triangles w.r.t. to the direction of observation.
(3) *CropNorm*—removes triangles that are oriented unfavorably to the sensor.
(4) *CropNear*—applied after the RT analysis, this filter removes the triangles that are too close to the sensor. The occlusions generated by these triangles are still considered in the analysis, but their presence is then filtered out.
(5) Further filtering for 3D (RGB-D) cameras—in case the devised system consists of 3D optical sensors, a further filter has to be applied to take into account the presence of two devices (i.e., camera/projector) contemporarily interacting with the scene.

Without the filters, every configuration with a sensor not observing the scene at all would require a cycle on each STL triangle to assess its visibility. The pre-processing filters allow the identification of “empty” configuration a-priori, saving a lot of resources. As stated above, each filter is described in detail in the following sub-sections.

##### CropFOV

**Figure 3:**
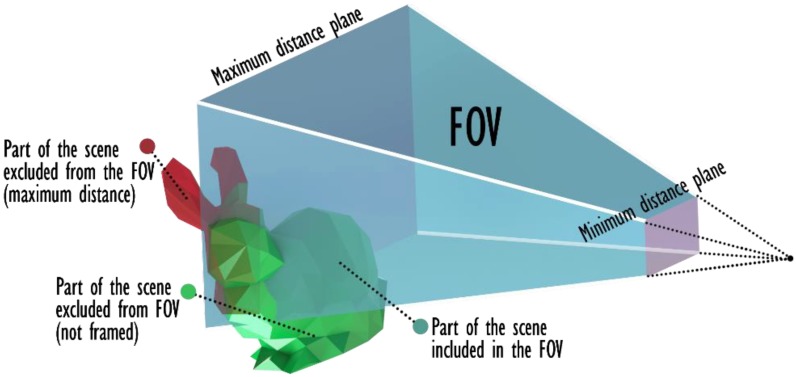
Effect induced by the *CropFov* filter.

The viewing frustum is described as a truncated pyramid (Figure 3) with a rectangular basis, whose shape is defined by two angles (α, β) that describe the horizontal and vertical aperture. The pyramid is limited at the far extremity by a plane positioned at maxD distance along the optical axis. The *CropFOV* filter removes all the triangles falling outside the FOV volume. Areas that are too close to the sensor are, in this phase, still maintained intact since they could generate occlusions that need to be considered.

The intersection between the viewing frustum and the scene model is computed by means of a convex hull algorithm. MATLAB® *inhull* algorithm is used to isolate all the points of the model that fall within the FOV volume. The original triangulation is then pruned from the triangles that have all vertices among the removed ones. As a result, some triangles that are partially located outside the FOV are left by the filter, but this effect is tolerated. Depending on the application, the average dimension of the triangles might be monitored in order to keep this and similar effects under control. 

##### CropBack

**Figure 4:**
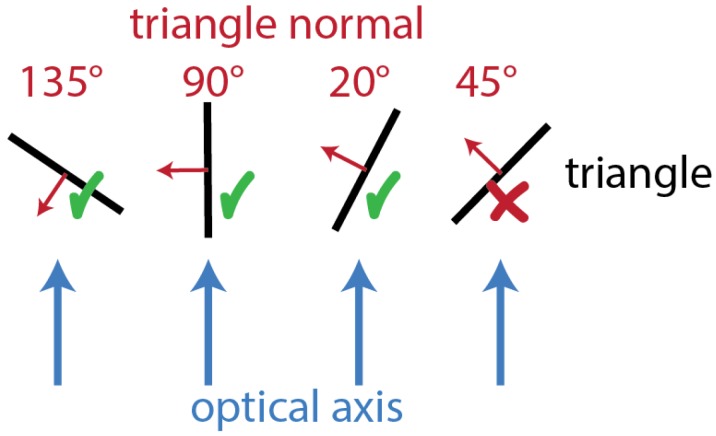
Effect of the *CropBack* filter.

Back-faced triangles are oppositely oriented with respect to the sensor; therefore, they are not visible even if they are lying in the viewing frustum (most of them are actually occluded by other triangles). Their identification can be carried out by means of a simple dot product between the vector of the optical ray hitting the triangle and the normal of the triangle itself. This implementation, however, would require the computation of a series of vectors that are directed towards each specific triangle. A faster way is to make use of the sensor optical axis instead of each optical ray. Some uncertainties are introduced by following this approach since, depending on the angles characterizing the FOV aperture, a sensible difference could be introduced. This effect could bring both to false positive and false negative. Considering the nature of this step, which aims at providing a filter for subsequent operations, the problem is solved by introducing a threshold value which tolerates back-faced triangles up to a certain orientation (set to 20° in the present work). The value can be tuned to satisfy different specific needs, depending on the application. The effect introduced is visible in Figure 4. While the removal of backfaced triangles could occasionally lead to the misinterpretation of occlusions on the scene, this occurrence is really limited; moreover, this situation is triggered by low quality scene models, condition that should be nonetheless monitored. 

##### CropNorm

This step is a further extension of the previous *CropBack* filter. The same result (i.e., orientation of the triangle w.r.t. the sensor) can be used also to perform an additional filter to cull triangles that are oriented in a direction that does not favor their correct acquisition. While they are not back-faced, their normal vectors are almost orthogonal to the optical axis of the camera. Accordingly, such triangles offer only a minimal portion of their surface to the observer, i.e., their projection on the direction of observation. This filter is generally optional as it introduces an effect that is difficult to calibrate correctly (otherwise there would be no need of the previous one). However, it is essential dealing with 3D sensors, which are heavily affected by surface inclination w.r.t. the sensor. As a result, *CropNorm* can simulate the physical behavior of a sensor able to acquire a surface only up to a certain orientation. The filter can be applied in a simplified version, which makes use of the optical axis as the only direction considered in the evaluation of the triangle inclination. Alternatively, the exact directions could be computed (tracing vectors from the camera origin to the center of mass of each triangle) and used for the estimation of exact angles. This operation, however, increases the computational cost of this filter: accordingly, its introduction is optional and its activation left to the user. 

The identification of a standard threshold value to be used in the *CropNorm* filter is not possible: the angle needs to be determined according to the performances of the sensor. Physical tests are also recommended to compare the results provided by the GUI with the actual scanning performances of the sensor.

##### CropNear

**Figure 5:**
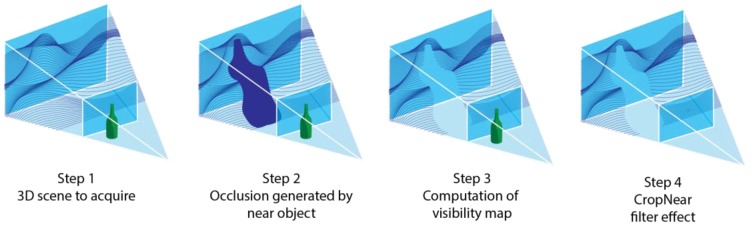
Steps of the process leading to the *CropNear* filter.

The triangles found by the RT algorithm need to be processed through a final step to remove those that are too close to the sensor. These should be accounted for possible occlusions but they should not be counted as visible not to bias the global metric. Accordingly, it is applied after the RT algorithm, as shown in Figure 2. The effect caused by this filter is depicted in Figure 5: the presence of near objects causes occlusions on the scene that need to be considered (step 2). Once that the VM is computed (step 3), the near objects can be removed from the final VM (step 4—*CropNear* filter). 

On a practical level, the filter is applied similarly to the *CropFOV*, by evaluating which points of the model fall outside of the acquisition zone and removing the triangles that are not entirely contained in the viewing frustum. When dealing with sensors that are not affected by a minimum acquisition distance the effect of the filter is removed by setting minD to zero. 

##### Further Filtering for 3D (RGB-D) Sensors

**Figure 6:**
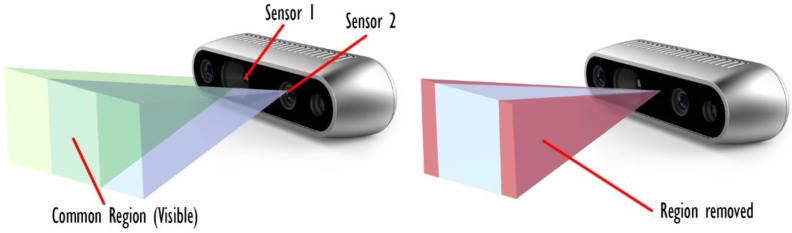
Modelling of the Field of View (FOV) of triangulation-based sensors.

**Figure 7:**
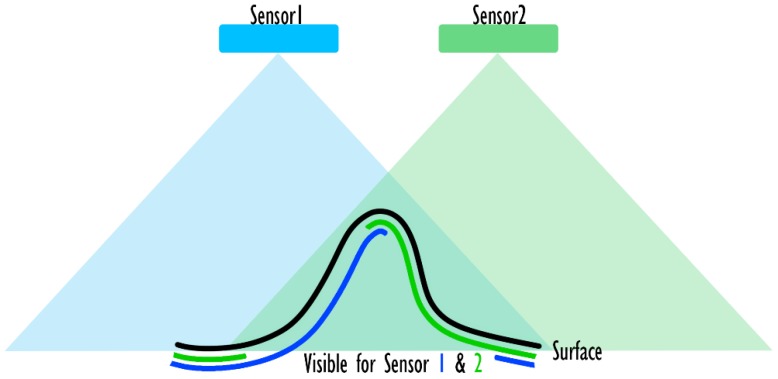
Modelling of the FOV of triangulation-based sensors: effects introduced by the intersection of occlusions.

**Figure 8:**
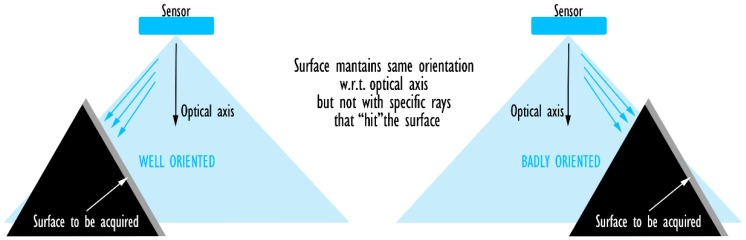
Modelling of the FOV of triangulation-based sensors. Effects of the relative position and orientation of a surface w.r.t. the sensor observing the scene. Use of optical axis vs actual optical rays.

When the architecture to be devised is based on a 3D (RGB-D) sensor, another important aspect needs to be considered to perform the visibility analysis: the modeling of the sensor itself. In fact, such sensors make use of two different devices to acquire stereographic information on the scene. Such sensors cannot be exactly modeled using a pinhole camera model, which makes use of a perspective projection that relates all the element on the scene to a single point. Indeed, this hypothesis is not valid in case of triangulation-based sensors (e.g., Figure 6), that produce a VM that should be described as the intersection of two pinhole camera models. However, considering a small approximation, is possible to simulate VMs of 3D optical sensors with a single pinhole camera model, positioned in the center and with a slightly reduced viewing frustum (Figure 6). FOV angles are usually provided by the producers of RGB-D cameras and can be used directly to build a valid model (at least for the shape of the pyramidal FOV). Unfortunately, the intersection effect is not only limited to the shape of the FOV, as every point need to be visible by both sensors/emitters for it to be acquired. This means that the model of the sensor needs to account for situations like the one depicted in Figure 7. Although the surface to be acquired is included in the volumes of both FOVs, due to its shape it is only marginally acquired by the sensor. The only surfaces acquired are the ones highlighted both in green and blue. The areas that are most affected by this phenomenon are the surfaces that are close to orthogonal w.r.t the camera axis. Accordingly, the solution implemented in this method relies on the application of the *CropNorm* filter to further remove triangles that are badly oriented. This solution is not exact, as it should account for the specific direction of the ray observing the triangle rather that considering the optical axis of the sensor to be more accurate (see Figure 8 for an exaggerated representation of the possible errors caused by this approximation) but it is an acceptable approximation.

#### 2.1.2. RT Analysis and Visibility Map

The RT analysis is performed using a GPU implementation [13]. Depending on the hardware available to the final user, different performances can be achieved in the computation of the VM; specifically, the GPU implementation poses a maximum number of optical rays that can be evaluated by the system; this is due to the fact that the implementation relies on the parallelization in the computation of each optical ray, up to the maximum allowed by the number of GPU cores. The traditional implementation, although generally slower, does not pose a rigid upper bound to the number of rays considered in the analysis and could be a valid alternative in some cases. Two different methods can be used to trace optical rays and evaluate a complete VM. 

The first one uses the image plane of the sensor as reference to create a series of optical rays originating from the camera optical center and “sweeping” the image plane, discretizing it with a given angular step. Such a step has to be small enough to either (1) create a series of rays intersecting at least one time every triangle in the model or (2) simulating the acquisition resolution of the sensor. In both cases, a high number of rays can be originated, depending on the triangles size and distribution within the FOV. 

The second method uses the triangles composing the model as reference (the coordinates of the vertices composing the triangles are known). Since the previous filtering steps reduced the scene model to the triangles included in the FOV, is possible to cycle through them to generate the exact number of optical rays, each one hitting the triangle in the center of mass.

The second strategy is used in this implementation. Accordingly, all the centers of mass of the remaining triangles are computed. Subsequently, the algorithm evaluates the direction of a series of vectors starting in the camera origin and directed towards each center of mass. These are fed to the RT algorithm to compute the intersection with the triangles, consider occlusions effect and build the final *VM.* By following this strategy is possible to limit the amount of resources used by the algorithm, to tailor the algorithm to the characteristics of the scene model (average area of triangles and their position w.r.t. the FOV). Depending on the number of triangles included in the viewing frustum, the difference in the time required by the two approaches could be significant: dealing with a viewing frustum that is almost empty, the required time is practically instantaneous.

It is noteworthy that this strategy could lead to the inclusion, among the triangles judged as visible, of elements that are only partially contained in the FOV; the incidence of this factor is directly related to the dimension of the triangles composing the model. If a higher accuracy is required, the “sweeping” strategy can be used to strictly guide the visibility analysis.

**Figure 9:**
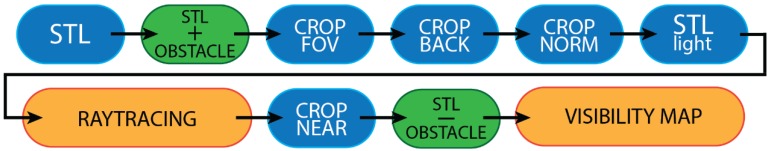
Visibility analysis framework: introduction of the element obstructing the scene (green blocks).

In order to increase the pool of possible application scenarios for the presented procedure, the presence of an “obstacle model” was included in the method. This scenario considers the presence on the scene of a series of surfaces and solids that, even if detectable and observable by the sensors, are not of interest to the user. In other words, their presence is not beneficial for the acquisition and they should not be among the surfaces to frame and acquire. However, when dealing with this type of surfaces, the occlusions caused by their presence still needs to be considered. Accordingly, if an obstacle model is provided, the method adds it to the scene model at the start of the visibility analysis phase (first green element in Figure 8), performs all the steps previously described, and removes the part of the obstacle that resulted visible at the end of the process (second green element in Figure 9).

The formulation used to describe the final *VM* relies on the original triangle matrix of the STL; such matrix is modified by assigning a flag value to every triangle that is visible for the considered camera. Accordingly, the matrix can be filtered to extract only the observed triangles. 

### 2.2. Optimization Phase

The optimization procedure starts with a set of user-supplied parameters describing a plausible configuration for the sensors. This set is used as starting solution in the PSO, as it is included in the first population of solutions to be tested (named *InitialSwarm*). The rest of the *InitialSwarm* is created analyzing upper and lower bound that define the solution space and creating a set of solutions that are evenly spread across the space. Upper and lower bounds are provided by the user with reference to the specific problem faced and the desired domain for the extrinsic parameters.

Every solution corresponds to a configuration of the sensors; each sensor is tested applying the visibility analysis procedure to obtain a VM. This type of data describes which areas of the model are visible for the sensor. However, the PSO needs a valid metric to guide the optimization. A numeric value needs to be extrapolated from each of the n *VMs*. 

Moreover, every application might have an optimal function that is specifically suited to describe a problem. In this study, several functions of general validity that could be used if a specific formulation is missing when dealing with multiple sensors are here provided. 

#### 2.2.1. Objective Function

In the most straightforward implementation, the objective functions are applied to a single scene model. However, an interesting application scenario is the identification of the best positions for the observation of a series of scenes/objects. A typical example could be the design of an optical 3D scanner (e.g., a photogrammetric body scanner or a triangulation-based scanner). In this case the scanner should be able to perform well on a series of models that could be similar or even really different. The method proposed in this paper allows the computation, for each evaluation called by the PSO, of the chosen *objective function* on a series of STL models. This way, the resulting objective function is computed as an average value across all models. As a result, the optimization pursues a “global” result, avoiding the selection of parameters that could be valid only for a specific situation. This could be applied for the evaluation of the effects of possible occlusions or to test different scene configurations.

In the following, some objective functions that can be used to setup the system are described. 

##### Objective Function #1—Maximize Observed Area 

The function keeps track of all the triangles seen by all the sensors, maximizing the global number of elements observed. Each triangle contributes as a single unit, independently of how many sensors observe it, its surface area, the orientation from which is observed. As a result, the sensors are guided to take extrinsic parameters that cover the entire scene. Overlapping between sensors observed regions is neither stimulated nor discouraged. OF1 can be normalized by dividing the calculated value by the total number of triangles composing the scene model.

OF1 requires a basic comparison between all the n *VMs* to identify triangles that are seen by multiple sensors in order to count them only a single time in the global metric. Accordingly, the resulting function is described by Equation (1), where $\bar{T}$ (Equation (2)) is the set of triangles resulting from the union of n sets of triangles observed by the n sensors. $T_{scene}$ is the total number of triangles of the scene.

(1) $$OF1=\frac{card\left(\bar{T}\right)}{T_{scene}}$$

(2) $$\bar{T}={⋃}_{i=1}^{n}T_{i}$$

An alternative formulation of this objective function provides a metric evaluated considering the actual surface of each triangle. In this formulation, the value of each triangle within OF1 is not the same, but depends on its surface area; accordingly, the area of each triangle that is part of the VM is computed using the vertices coordinates and a cross product. This is significative only dealing with STL models that are characterized by triangles of different size and aspect ratios. In this case, a possible formulation for OF1 would be the one in Equation (3), where $a_{j}$ is the surface area of the j−th triangle (${\bar{t}}_{j})$ that is part of the “final” set $\bar{T}$.

(3) $$OF1_{area}=\frac{\sum_{j=1}^{card\left(\bar{T}\right)}{\bar{t}}_{j}a_{j}}{T_{scene}}$$

##### Objective Function #2—Maximize Observed Area and Overlap 

This OF considers the hypothesis of an application where a certain degree of overlapping between different sensors is encouraged in order to obtain redundant data. Moreover, dealing with 3D sensors, the data obtained from different sources needs to be aligned in order to reconstruct the whole surface. The ideal solution that OF2 should describe, is represented by a scene that is totally observed by the sensors, while assuring a certain amount of overlap. It is important to note that the function should not overly prioritize or stress the overlapping; in fact, the benefits introduced by multiple acquisition rapidly fade with the numerosity of observations of the same elements. In other words, there is little-to-none additional benefit in observing a triangles 3 or 7 times from different sensors. A notable exception might be the case of applications that need to consider the problem of moving elements: in this case the occlusions generated are different and difficult to control. As a result, a higher number of observations increases the probability of a correct acquisition of an area.

OF2 performs the same type of comparison of OF1*,* counting how many sensors see the same triangle and stores this value in a matrix, associating it with the triangle itself. The composition of OF2 across n sensors is performed introducing a series of weights to discriminate between various solutions, according to Equation (4):

(4) $$OF2=\overset{n}{\underset{i=1}{\sum }}w_{i}*card\left({\tilde{T}}_{i}\right)$$

where ${\tilde{T}}_{i}$ is the set of triangles observed i times. The weights can be tuned according to the specific application, considering that no negative effect should be introduced with $T_{1}$ (in all the case studies tested by the authors $w_{1}$ was set equal to 1). The weights sets tested and proposed by the authors are reported in Table 1. The main difference between the two sets concerns $T_{1}$: triangles observed only once can be considered as a “neutral” condition or a behavior that positively influences the global metric; in other words, these can be tolerated or incentivized by OF2. 

##### Objective Function #3—Composition of Factors 

OF3 combines three effects to obtain a more stable and generic metric. This function is based on both OF1 and OF2, which are integrated in Equation (5) together with a new value $T_{tot}$.

(5) $$OF3=w_{tot}T_{tot}+w_{OF1}OF1+w_{OF2}OF2$$

(6) $$T_{tot}=\overset{n}{\underset{i=1}{\sum }}card\left(T_{i}\right)$$

$T_{tot}$ is the total number of triangles observed by all the sensors; such value does not consider if a triangle observed by a sensor is also observed by another one. OF3 integrates the three values to build a metric that tries to (1) guide each sensor to cover the maximum possible area of the scene; (2) maximize the diversity among the regions observed by different sensors; (3) assure overlapping regions. By changing $w_{tot}$, $w_{OF1}$ and $w_{OF2}$ it is possible to favor one aspect over the others. The values tested by the authors are reported in Table 2. 

The best result, with respect to the case study discussed in Section 3, were obtained using the first set. 

Other objective functions could be imagined and built to include different aspects into the optimization. As an example, soft constraints could be included to consider the quality of the acquisition within the function. Considering 3D sensors, this could be achieved by considering the inclination of the triangles w.r.t. the sensor not only in the filters, but also in the final function. For instance, orientations close to orthogonal could be privileged. Moreover, surface area of the triangles observed by a sensor could substitute the count proposed in OF1/OF2/OF3 when uneven triangulations are used as reference models. Considering 2D sensors, an effect that could be modeled in the future is the resolution of the optical sensor (as proposed in [9]), which should penalize the acquisition of objects and surfaces from a long distance. Summing up, three different formulations which offer a simple yet effective description for multiple application scenarios are offered; the study of more refined modeling effects, while interesting from an academic perspective, offer little advantages considering that each application is characterized by different needs. As a result, a more in-depth study on objective function is left to the final user of the method, who is required to tune and adapt the procedure to their application.

## 3. Results

### 3.1. Arm Scanner

As previously mentioned, the entire procedure was tested on a real application i.e., the development of a 3D scanner equipped with RGB-D cameras for the acquisition of the arm-wrist-hand human district. The scanner is the first step of an innovative framework for the production of 3D printed casts for the arm (the whole procedure is described in [14]). 

**Figure 10:**
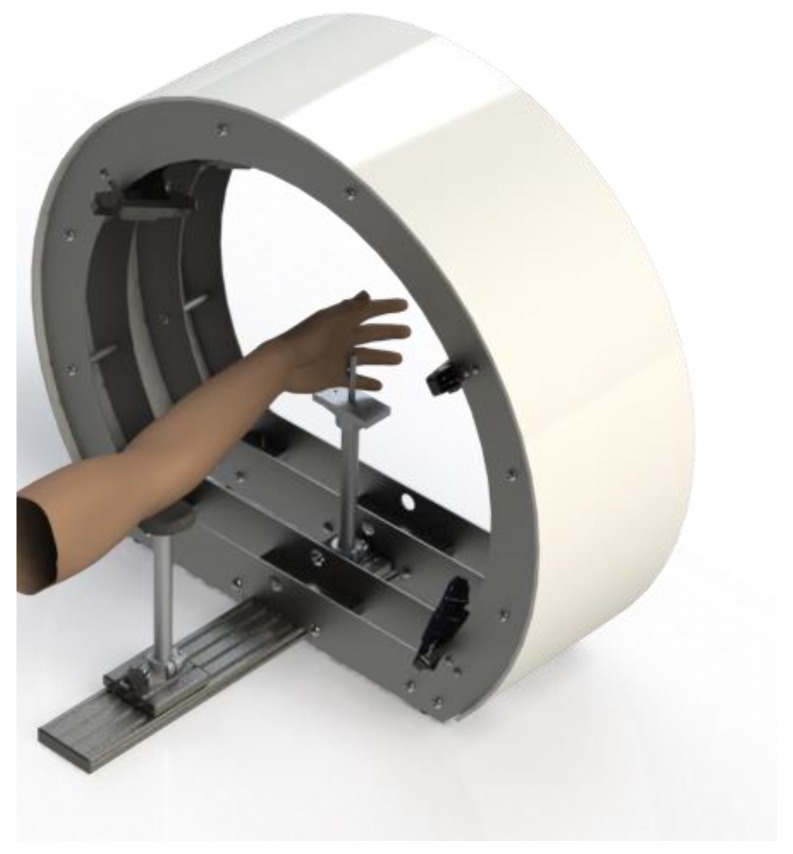
“Oplà” 3D scanner version 1.

The hypothesized architecture, based on two circular arrays of RGB-D sensors, allows the acquisition of the entire arm geometry in a very short time (lower than 0.5 s). The effectiveness of the chosen architecture was demonstrated during the development of a previous version of the scanner [15] (Figure 10), which used Intel SR300 cameras. The new version integrates a different kind of sensor (Intel’s D415 depth camera [16]) which allows improved performances w.r.t. the previous one. 

Some of the characteristics of the D415 depth camera are listed in Table 3. 

**Figure 11:**
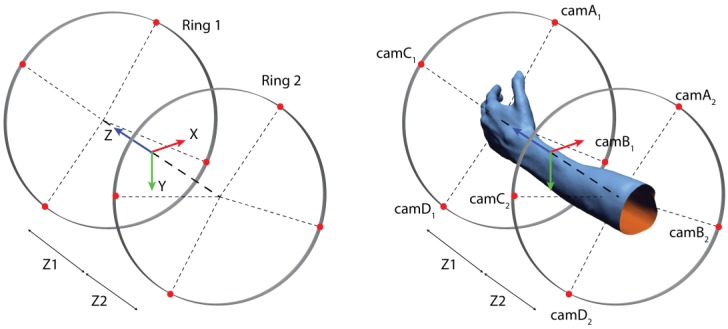
Optical sensors reference frame and coordinates.

As previously mentioned, the architecture of the scanner is based on two circular arrays of cameras observing the scene (i.e., patient arm). Eight cameras are used in the scanner: the number was determined after a rough evaluation of the FOV offered by each sensor. Therefore, the solution space for the optimization should consider this preliminary hypothesis. The model used to describe the solution space is depicted in Figure 11. 

The radial distance of each camera from the central axis of the scanner is set to a constant value and is evaluated a priori to maximize the quality of the data acquired by the sensors. The radius of the circular array was set to 250 mm referring to preliminary tests and consulting the specifications of the D415. Symmetry on the YZ plane is imposed for the disposition and orientation of the sensors in order to allow the acquisition of both left and right arms without any change in the configuration due to the different disposition of the main features of the target object. The Z-position of the two rings is to be determined by the optimization within a pre-established range. 

**Figure 12:**
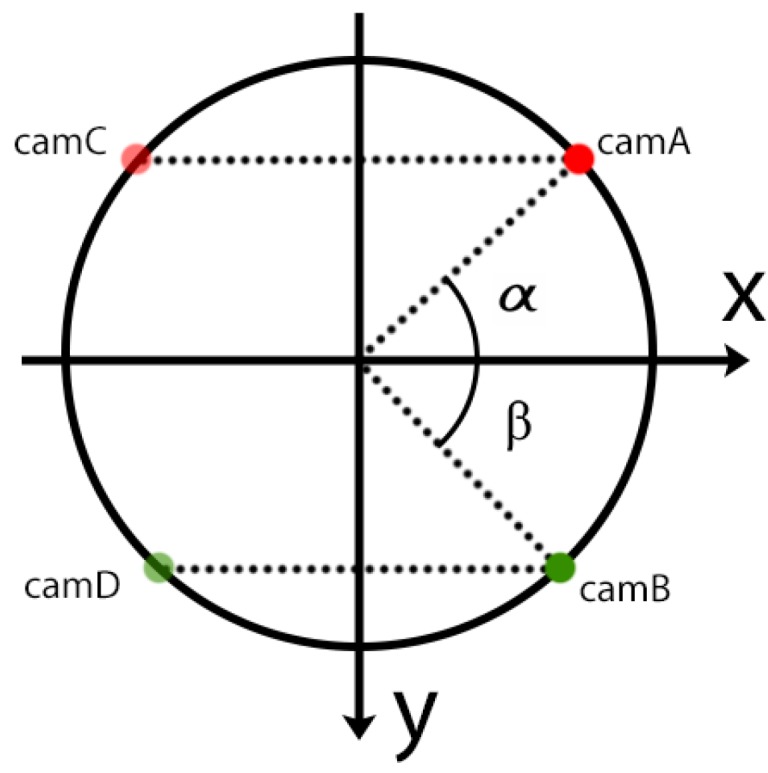
Coordinates defining the position of the cameras on a single ring.

On each ring, the position of the sensors is controlled according to the model depicted in Figure 12. The position on the circumference of two pairs of sensors, symmetric across the y-axis, is determined according to two angles α and β. The orientation of each pair of sensors is left free. 

As a result, the entire configuration is controlled by 18 parameters as reported in Table 4. Each parameter that pertains to the ring1 or ring2 is marked by using the subscript 1 or 2. The orientation of each camera is controlled by 3 variables as described in the previous Section (orientation and roll angle); the symmetry on the *YZ* plane is assured even for the orientation. The roll angle is expressed as a rotation around the optical axis according to the right-hand rule. In this case study a roll angle of 0° represents a camera with the mayor axis of the image plane coplanar with a plane parallel to the *XZ* plane.

To test the impact of the number of variables on the performance of the optimization and to evaluate the effect of roll angles into the procedure, a further optimization was run excluding the roll angles. This limits to 14 the number of parameters.

Plausible upper and lower bounds, reported in Table 4, were imposed for all the variables in order to guide the optimization in a valid region, ensuring manufacturability and usability of the device. The analysis was carried out on a pool of six STL models; accordingly, each solution is tested during the optimization procedure on each model. 

**Figure 13:**
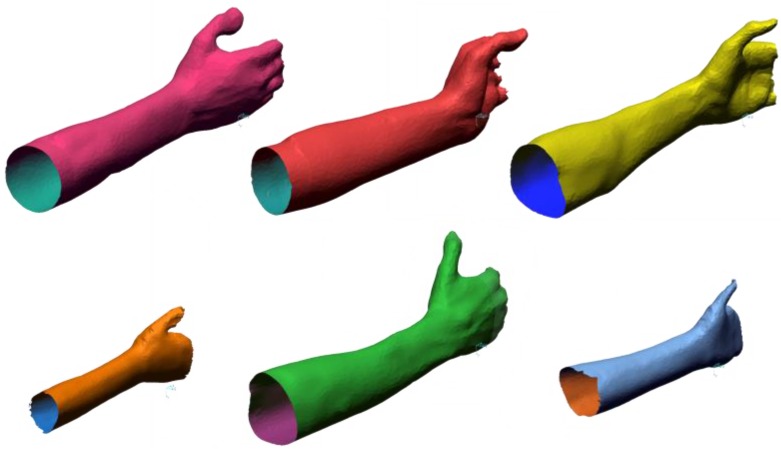
Arm models used in the analysis.

Consequently, the OF value is the average value computed across the six models. The models are generated using high-resolution 3D scans of arm-hand districts of both female and male subjects of different age. Both left/right arms were included in the set: all the arms were acquired in a similar position, but notable differences can be observed in local positions of the hands, as well as in the global anatomical characteristics (Figure 13). 

The STL models were processed in order to obtain a manageable number of triangles and to regularize their size. Regions that should not contribute to the analysis (e.g., top part of the fingers) were removed to not influence the computation of the *OF.* The models were aligned in order to match the reference system of the digital model with a known point of the scanner (i.e., the point where the hand should be placed during the acquisition of the arm). The average number of triangles is 8500, the average length of the triangles edges is 3.4 mm. Manual modelling operations were performed to amend local defects of the mesh (holes, inverted normal).

**Figure 14:**
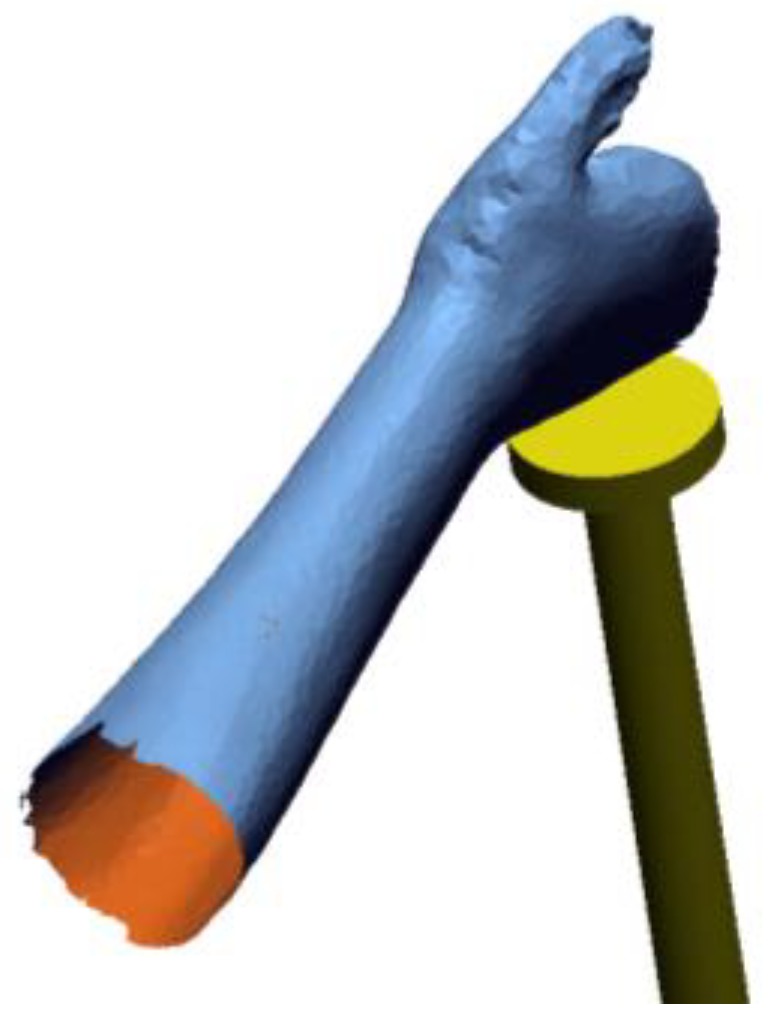
Arm model (blue) and hand support (yellow) correctly positioned. The occlusions generated by the obstacle (yellow) are considered in the analysis).

Occlusions from an obstacle object were considered in the analysis. The presence of the hand holder (i.e., the physical object required to support the hand in the right position, visible in Figure 9) was considered introducing an STL model as obstacle, as described in the previous section. All the hand models are correctly referenced to the hand support model as depicted in Figure 14. 

Table 5 shows the parameters used for carrying out the PSO. The performance of the optimization is shown in Table 6.

These results, as well as the final extrinsic parameters, were confirmed in a series of 10 tests repeated under the same conditions. The computation time of the VM is heavily affected by the configuration: a higher number of triangles placed in the area of observation of the sensors reduces the benefits of the filters and increases the time required to run the RT algorithm. Lowest values assessed around 0.08 s (on a single model) while the highest values are around 6 s (on a single model).

Referring to the final sensor extrinsic parameters, all optimization procedures provided results only slightly different one with each other (not relevant to the application under consideration, positions changed up to 1 mm total distance). Therefore, any of the obtained solutions can be used for defining the final architecture of the MV system. Table 7 and Table 8 show the average value obtained for the 10 tests. An increase in the computational resources offered to the algorithm didn’t brought significant changes in the results. 

**Figure 15:**
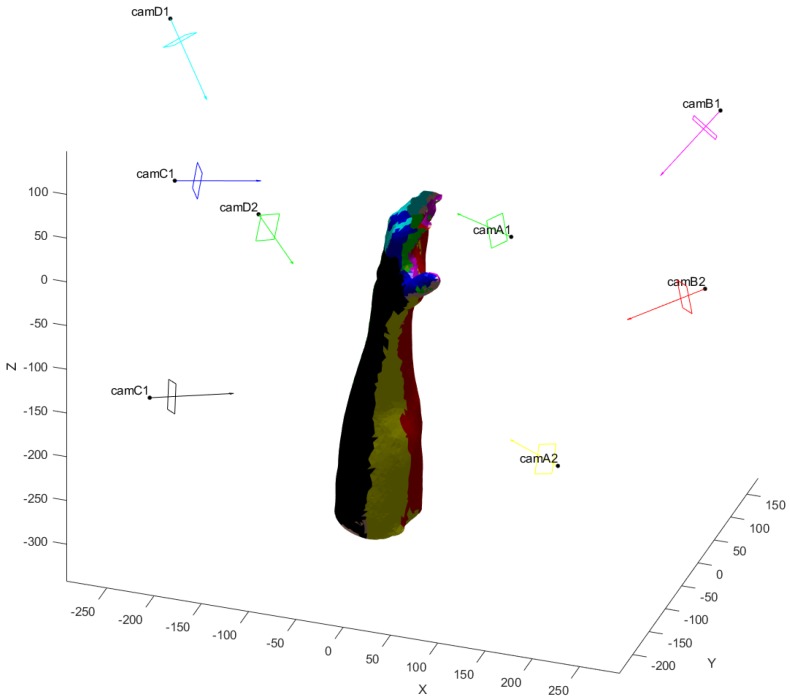
Final positions of the sensors with the visible color-coded regions on an arm model. Dimensions in (mm).

**Figure 16:**
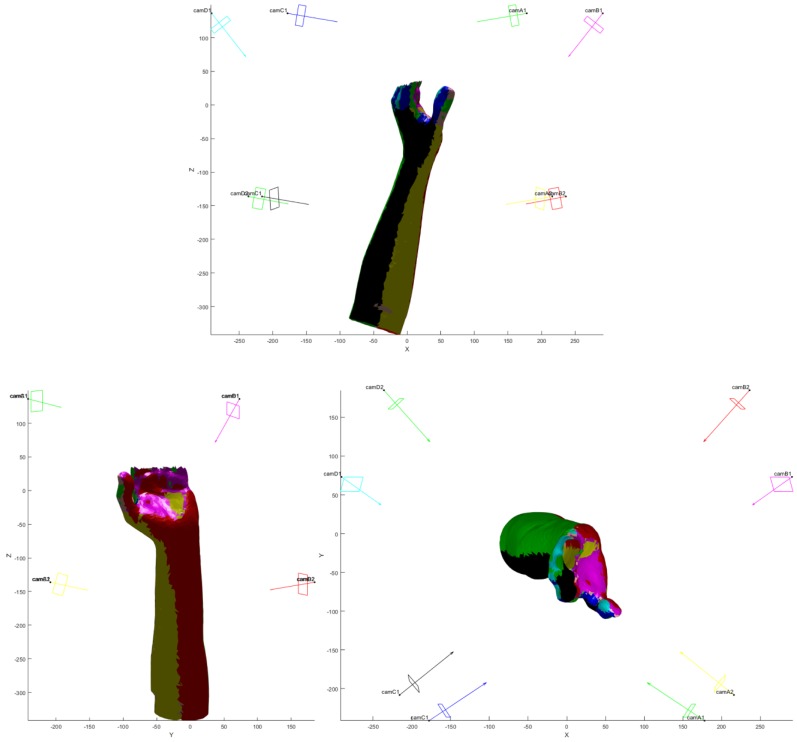
Final positions of the sensors with the visible color-coded regions on an arm model. Dimensions in (mm). Top, front and lateral views of the arm depicted in Figure 14.

The final VMs, visualized thanks to the appositely devised GUI, are in Figure 15 and Figure 16 for one of the models. Each color identifies a camera and the observed triangles. Each sensor is represented with optical center, image plane and optical axis to convey the information on its position and orientation to the user.

The values obtained assured a total visibility always higher than 97% for all the models considered in the analysis. Table 9 reports the result in detail: the total number of triangles observed for each model is reported, along with the percentage of visibility achieved with the final configuration. All the areas observed by the sensors were characterized by a significant amount of overlap, with a circa 40% of the model always covered by more than one camera. This amount of overlapping is valid for subsequent registration and data-denoising operations. 

The improved version of the scanner is capable of performance significantly different w.r.t. the original one presented in [14]. The most important element is the robustness guaranteed by the device w.r.t. different anatomies that need to be acquired. The tests performed on different dimensions and types of arms allowed the identification of extrinsic camera parameters that perform well across the entire range of possible patients to acquire (both in dimensions, right or left arm, specific anatomical features of the arm). The new hardware used to build the device performs well in the acquisition of any type of surface (even human skin, which is essential) and allows for a higher scanning resolution and acquisition. The orthopedic team of the Meyer Children Hospital of Florence has provided positive feedback on the tests performed with the scanner, which will be used in the next future in the context of a medical trial experimentation to treat different kinds of wrist fractures in young children. 

### 3.2. TOF Acquisition of An Indoor Environment 

**Figure 17:**
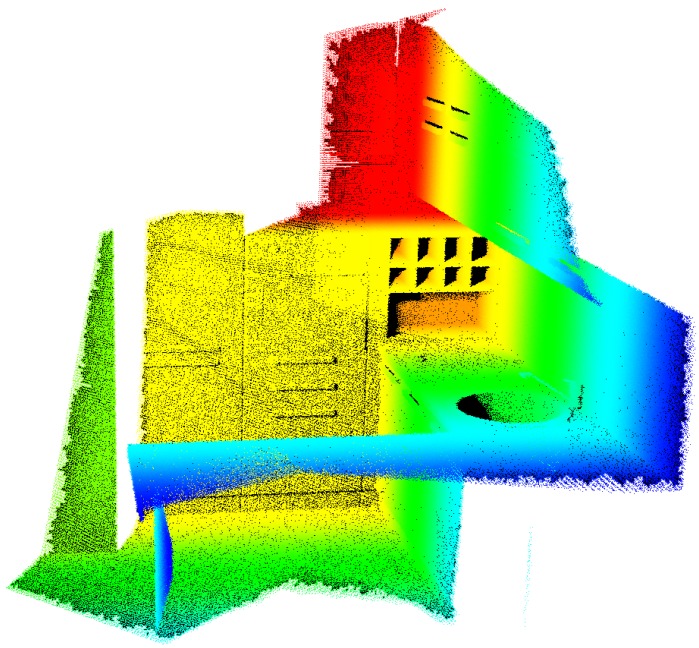
Original reference data extracted from the BlenSor database—kitchen model.

In order to increase the generality of the method, the procedure was tested on an additional case study: the setup of 2 depth sensors (i.e., Microsoft Kinect v2) observing an indoor environment. Literature parameters of the Kinect were used to model the cameras [18]. Digital synthetic data, extracted from the BlenSor database [19], was used as coarse model of the scene to run the analysis. The data refers to a set of kitchen furniture depicted in Figure 17, acquired by using a TOF scanner, positioned in multiple acquisition points. The original data was composed by 1775 k points, which were heavily reduced (to 3.5 k points) to build the coarse model describing the scene; regular dimension of the triangles was verified.

Two cameras were placed in the scene, constraining their optical centers to the volume of a hypothetical room (3 m × 3 m × 3.5 m) containing the scanned furniture. The simulation was run trying to maximize the number of triangles observed by the couple of cameras, hence not considering the need for overlapping regions. In this second case, a weight set characterized by $w_{tot}=1,\;w_{OF1}=10,\;w_{OF2}=0$ was hence used.

The parameter vector used in this case was composed by the extrinsic parameters of the two cameras, without any additional constraints limiting the optimization, resulting in 12 independent parameters (Equation (7)). The parameter vector was initialized with both cameras coincident in the same central point of the room, with orthogonal optical axes.

(7) $$x=\left[X_{1},\;Y_{1},\;Z_{1},X_{2},\;Y_{2},\;Z_{2},\;\theta_{1},\;\gamma_{1},\;\theta_{2},\;\gamma_{2},\;roll_{1},\;roll_{2}\right]$$

**Figure 18:**
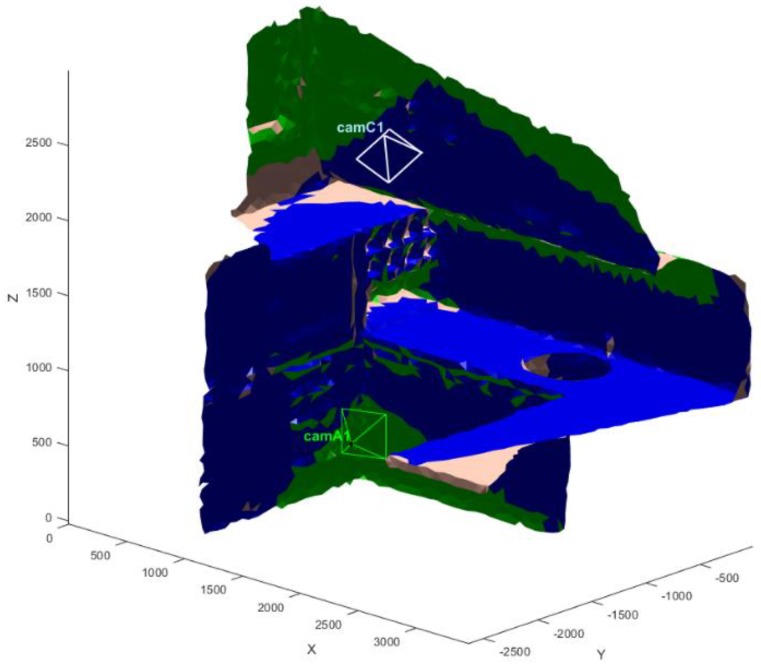
Final visibility results obtained with two sensors observing the BlenSor Kitchen model. The triangles observed by the first camera are colored in green; the triangles observed by the second camera are colored in blue.

With the exception previously mentioned, optimization settings reported in Table 5 were used for this case. Table 10 reports the final extrinsic parameters obtained at the end of the optimization. The reference system is placed on one of the corners of the room. Table 11 reports the visibility results of the analysis; the experiment has been executed 5 times and an example of the obtained results is depicted in Figure 18. 

**Figure 19:**
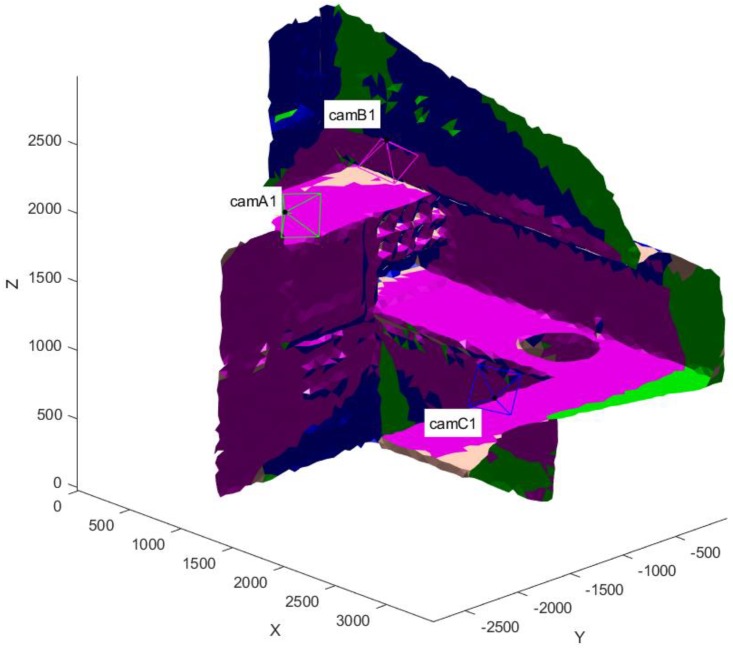
Visibility Map computed on the BlenSor kitchen model with three sensors observing the scene. The triangles observed by the first camera are colored in green; the triangles observed by the second camera are colored in blue; triangles observed by the third camera are colored in magenta.

**Figure 20:**
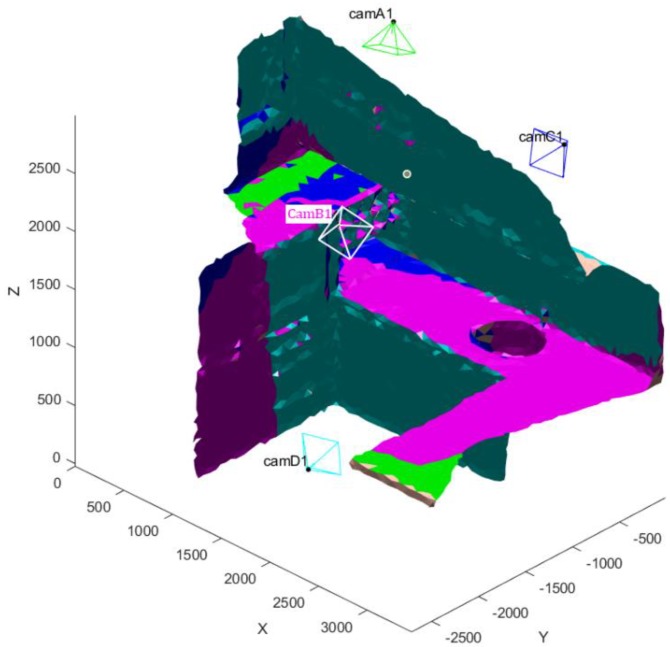
Visibility Map computed on the BlenSor kitchen model with four sensors observing the scene. Triangles observed by the first camera are colored in green; the triangles observed by the second camera are colored in blue; triangles observed by third camera are colored in magenta; triangles observed by the fourth camera are colored in cyan.

The analysis was subsequently carried out with an increasing number of cameras to test how visibility improves; the results were characterized by a higher variability in the position of the sensors obtained at the end of the optimization w.r.t. previous results discussed in the text. This is probably due to the fact that no constraints on the extrinsic parameters were imposed, resulting in a problem with multiple equally valid solutions. Accordingly, the configurations identified by the method do not define a unique set of position for the cameras, although the results in terms of visibility are comparable. This is confirmed considering the results for the number of unique triangles observed by the cameras, which was the main factor driving the optimization. Examples of the results obtained with three and four sensors are depicted in Figure 19 and Figure 20. The results in terms of triangles observed are summarized in Table 11. 

The procedure developed proved to be highly customizable to the specific application needs, as quick changes of the weights, camera intrinsic parameters and constraints imposed on the extrinsic parameters allow for the achievement of different results in short times.

## 4. Discussion and Conclusions

A procedure to perform an optimization search for the best extrinsic parameters of a set of optical sensors observing a known scene has been presented in this article. The method can model both 2D and 3D sensors, thanks to an updated version of the classic pin-hole camera model, which is processed with a series of filters to assure i) good computational performances of the model, ii) a valid representation of triangulation-based optical 3D sensors. The development of an optimization procedure for visibility analyses of 3D optical sensors, specifically, represents a significant contribution to the state of the art. The method is based on a PSO algorithm performing the seek for the best set of parameters. The features and performances of the devised method have been discussed referring mainly to the development of a 3D scanner built with the integration of the data acquired by eight RGB-D cameras. The method allowed the identification of valid extrinsic parameters of the sensors, providing a starting point for the mechanical design of the device. Future work on the optimization procedure could be oriented towards the introduction of more advanced elements in the models used to describe the sensors (e.g., penalization factors for the quality (accuracy/resolution) of acquisition depending on the distance from the sensors). Such aspect is important whenever the application requires a precise evaluation of the accuracy or the resolution of the acquire data. Most times, however, the user is aware of a “safe zone”, w.r.t. the distance of acquisition, that guarantees sufficient data quality for the specific application faced. Accordingly, the presented model allows the definition of a custom viewing frustum that includes only the data of interest (i.e., points that fall within a certain distance range). As discussed in the text, more complicated models could take into account shininess and ambient illumination effects of data degradation, but these more advanced factors falls outside the scope of the present paper. Alternatively, efforts could be spent to build a database of validated models of most common 2D and 3D sensors. This could allow the expansion of optimization and also the identification of the ideal sensor and increase the usability of the whole procedure. 

## Figures and Tables

**Table 1:** OF2 weights tested in the study.

#WeightSet	$w_{1}$	$w_{2}$	$w_{3}$	$w_{4}$	$w_{5}$	$w_{6\ldots k}$
1	1	10	10	−2	−4	−8
2	10	10	10	−5	−10	−10

**Table 2:** OF3 weights tested in the study.

#WeightSet	$w_{tot}$	$w_{OF1}$	$w_{OF2}$
1	1	2	1
2	1	2	3

**Table 3:** Parameters used to model the behavior of the D415 camera in the study.

Parameter	Value
Minimum acquisition distance *	160 mm
Maximum acquisition distance *	350 mm
Resolution	1280 × 720
FOV	69.4° × 42.5° + (−3°) (conservatively set at 66.4° × 39.5°)
Maximum inclination of the triangles *	70° (0° being with the normal directed towards the optical center of the sensor and 90° being a triangle with normal orthogonal to the local optical ray)

* values that are valid for the specific application, they were estimated during experimental tests.

**Table 4:** Parameters used in the case study to model the solution space for the eight sensors.

Parameter Name	Parameter Effect
$Z_{1},\;Z_{2}$	Z position of ring1 and ring2
$\alpha_{1},\;\beta_{1},\alpha_{2},\;\beta_{2}$	Angular position of the sensors on ring1 and ring2
$A_{1}or_{I},\;A_{1}or_{II}$	Orientation of the camera A on ring1
$B_{1}or_{I},\;B_{1}or_{II}$	Orientation of the camera B on ring1
$A_{2}or_{I},\;A_{2}or_{II}$	Orientation of the camera A on ring2
$B_{2}or_{I},\;B_{2}or_{II}$	Orientation of the camera B on ring2
$rollA_{1},\;rollB_{1},rollA_{2},\;rollB_{2}$	Roll angles for sensors A&B on ring1&2

**Table 5:** Particle Swarm Optimization (PSO) settings [17] used in the analysis.

Parameter	Value
SwarmSize	300
MaxIterations	200
ObjectiveLimit	0
MaxStallIterations	30
InertiaRange	[0.2 1.5]
InitialSwarmSpan	20
FunctionTolerance	0.001
MinNeighborsFraction	0.4
Objective Function used	minimization of $\left(\frac{1}{OF\#3}\right)$, weight sets #1
Starting configuration	Cameras at 45°, observing the z-axis, roll 0°

**Table 6:** Optimization procedure details.

	Total Time [hours]	#Iterations	Avg. Time Per Function Evaluation (on All Models) [s]
With roll	41.3 h	76	6.44

**Table 7:** Cylindrical coordinates of the camera positions on the two circular arrays.

		Z [mm]	A [degrees]	β [degrees]
With roll	Ring 1	136	54°	14°
	Ring 2	−136	44°	38°

**Table 8:** Cartesian coordinates of the camera positions, direction of the optical axis expressed as a unit vector, roll angle of the cameras.

	Camera	X [mm]	Y [mm]	Z [mm]	Direction	Roll [deegres]
With roll	$camA_{1}$	178	−242	136	[−0.82 0.55 −0.14]	0°
	$camB_{1}$	291	73	136	[−0.57 −0.40 −0.72]	0°
	$camC_{1}$	−178	−242	136	[0.82 0.55 −0.14]	0°
	$camD_{1}$	−291	73	136	[0.57 −0.40 −0.72]	0°
	$camA_{2}$	216	−208	−136	[−0.77 0.62 −0.13]	20°
	$camB_{2}$	236	185	−136	[−0.66 −0.74 −0.12]	0°
	$camC_{2}$	−216	−208	−136	[0.77 0.62 −0.13]	−20°
	$camD_{2}$	−236	185	−136	[0.66 −0.74 −0.12]	0°

**Table 9:** Visibility results: triangles observed by all cameras, across each model. Both unique and total triangles observed are provided.

Model	Triangles Observed by All Cameras	Unique Triangles Observed	Total Triangles of the Model	Visibility Percentage
1	13,062	7867	7985	98.5%
2	14,613	8254	8341	99.0%
3	13,790	8647	8767	98.6%
4	13,508	8590	8726	98.4%
5	12,925	8632	8830	97.7%
6	12,487	8198	8409	97.5%

**Table 10:** Extrinsic parameters identified by the analysis as global optimum for the BlenSor kitchen model; Data refers to configuration with two sensors.

$X_{1}$	$Y_{1}$	$Z_{1}$	$X_{2}$	$Y_{2}$	$Z_{2}$	$\theta_{1}$	$\gamma_{1}$	$\theta_{2}$	$\gamma_{2}$	$roll_{1}$	$roll_{2}$
2567	2485	1293	2449	2047	3000	2.00	6.22	2.12	5.32	1.70	1.59

**Table 11:** Visibility results in terms of triangles observed with an increasing number of sensors observing the scene.

Number of Sensors	Triangles Observed by All Cameras	Unique Triangles Observed	Total Triangles of the Model	Visibility Percentage
2	6882 [321]	5198 [66]	6500	80%
3	8163 [1535]	5412 [120]	6500	83%
4	9769 [1152]	5823 [90]	6500	90%
